# Carbon monoxide and other “tricks” in sports: does the winner take it all?

**DOI:** 10.3389/fspor.2026.1847660

**Published:** 2026-08-14

**Authors:** Ilkka H. A. Heinonen

**Affiliations:** 1 Turku PET Centre, University of Turku and Turku University Hospital, Turku, Finland; 2 The UKK Institute for Health Promotion Research, Tampere, Finland

**Keywords:** active living, athletes, carbon monoxide, performance, physiology, safety, winning, youth sports

## Introduction

In the relentless pursuit of success and fame, many athletes and teams are willing to exhaust nearly all possible means to gain a competitive edge, which was seen also during the 2026 Milano-Cortina Winter Olympics. While this may include even doping, it more commonly involves exploiting technological innovations and advanced training techniques (1–4), which have likely contributed—at least in part—to the consistent progression of world records over the past century (5). One such approach, particularly in endurance sports but also gaining traction in team sports, is altitude training. Despite its popularity, its efficacy remains contested (6). Nevertheless, hypoxic chambers and other means to artificially stimulate hypoxic conditions are now allowed in most nations (7). Few months before the Olympics global media headlines wrote (8–13) that a Norwegian biathlon athlete was found dead in his hotel room during the altitude preparation training camp, with a hypoxia mask on his face. This tragedy illustrates that some athletes may be willing to adopt increasingly demanding methods in pursuit of performance gains.

## Carbon monoxide – many functions and roles

Within this highly competitive culture, athletes may feel compelled to adopt any method perceived to provide an advantage, regardless of whether robust scientific evidence supports its effectiveness and safety. A recent controversial method drawing attention to athletes is the repeated inhalation of carbon monoxide (CO), with some studies suggesting a potential increase in hemoglobin mass and exercise performance (14–17).

Many prominent scientists, physicians, and exercise physiologists have argued that CO inhalation is artificial, hazardous, ethically questionable—and in some jurisdictions, illegal (18–21). The International Cycling Union has already banned its use, and the World Anti-Doping Agency followed, based on current regulations and concerns over athlete safety. Although repeated inhalation of CO is now banned, this was previously unclear and the athletes used it to try to get a small effect. This being the case, in this opinion article CO is only used as an example of the actions and all possible methods that athletes are seeking to improve their performance, and to highlight its other functions. An importantly point to be emphasized here is also that rigorous scientific investigation on this and similar practices is important to enable evidence-based decisions. For instance, science has played a crucial role in banning blood doping (22, 23) and erythropoietin (EPO) (24, 25) by elucidating their performance-enhancing and physiological effects. In a similar way, studies on the effects of repeated CO inhalation are ongoing (26).

Importantly, CO is not intrinsically harmful. Like nitric oxide, CO is endogenously produced in the human body, acts as a signaling molecule, and may have therapeutic applications (27, 28). It has been extensively used to measure hemoglobin mass and blood volume—techniques that have significantly contributed to our understanding of physiological responses to altitude and other stimuli (29). In small, radiolabeled quantities, CO can also be used to quantify blood volume in specific tissues, such as the myocardium, which would otherwise be difficult to assess (30).

However, due to CO's strong binding affinity for hemoglobin—impairing oxygen transport and inducing hypoxia—it can also trigger EPO production similarly to altitude exposure or smoking (31). This same mechanism also poses serious risks, including the potential for systemic inflammation and toxicity with repeated exposure (32). Furthermore, even with repeated administration, CO may not yield measurable performance gains due to complications due to aforementioned reasons, as seen with the mixed results from hypoxia chamber use that do not always lead to increases in fitness and performance despite of increased hemoglobin mass (33).

Although CO is a naturally occurring molecule, particularly its historical associations inevitably also warrant caution. Most notably, it was used by the Nazi regime during the Aktion T4 euthanasia program, which preceded the mass killings of the Holocaust. Against this historical backdrop, the description by Minson and Joyner of CO use for performance enhancement as “dancing with the devil” is particularly thought-provoking (19), a perspective that the sport community should be aware of.

## Further perspectives

Viewed more broadly, neither CO inhalation nor any other performance “shortcut” can substitute for the foundational adaptations required to achieve elite athletic performance. True success stems from years, even decades, of consistent, intelligent training that develops cardiovascular capacity and other physiological and performance traits (34, 35).

Interestingly, while sports leaders often portray athletes as paragons of fair play and role models for youth, this perception may be overly idealistic as the reality is more complicated. Studies suggest that over 50% of elite athletes would consider using doping if it guaranteed success—even if it meant death within five years. The CO inhalation approach is a good example of that attitude. This “Goldman Dilemma” starkly contrasts with attitudes in the general population, where almost no one would accept such a trade-off (36). Moreover, data show that Olympic gold medalists have shorter lifespans than silver medalists, suggesting that the relentless pursuit of winning can come at a profound personal cost (37).

Perhaps greater recognition should be given to the many athletes who do not win. This is especially relevant in youth sport, where enjoyment, participation, healthy development, and lifelong engagement in physical activity should remain central objectives (38). Without the vast majority of competitors who do not stand on the podium, competitive sport would lose much of its meaning. Moreover, many of these athletes may lead more balanced lives, less influenced by the pressures that can promote increasingly risky performance-enhancing practices. They may better embody the values we claim to admire in sport: resilience, integrity, and commitment, enjoyment and simply active living which should be particularly at the population level far more important than anything else.

Ultimately, true champions are those who believe in and adhere to a well-structured, evidence-based training regimen. They continually refine their approach, guided by emerging scientific knowledge in exercise physiology, nutrition, and recovery (39, 40). Ironically, in my opinion those who “do not take it all” in the literal sense may end up taking the greatest rewards and achieving the most—enduring success, longevity, and respect – both in sport and in life.
